# Spatial Hearing with Incongruent Visual or Auditory Room Cues

**DOI:** 10.1038/srep37342

**Published:** 2016-11-17

**Authors:** Juan C. Gil-Carvajal, Jens Cubick, Sébastien Santurette, Torsten Dau

**Affiliations:** 1 Hearing Systems, Department of Electrical Engineering, Technical University of Denmark, Ørsteds Plads 352, 2800, Kgs. Lyngby, Denmark

## Abstract

In day-to-day life, humans usually perceive the location of sound sources as outside their heads. This externalized auditory spatial perception can be reproduced through headphones by recreating the sound pressure generated by the source at the listener’s eardrums. This requires the acoustical features of the recording environment and listener’s anatomy to be recorded at the listener’s ear canals. Although the resulting auditory images can be indistinguishable from real-world sources, their externalization may be less robust when the playback and recording environments differ. Here we tested whether a mismatch between playback and recording room reduces perceived distance, azimuthal direction, and compactness of the auditory image, and whether this is mostly due to incongruent auditory cues or to expectations generated from the visual impression of the room. Perceived distance ratings decreased significantly when collected in a more reverberant environment than the recording room, whereas azimuthal direction and compactness remained room independent. Moreover, modifying visual room-related cues had no effect on these three attributes, while incongruent auditory room-related cues between the recording and playback room did affect distance perception. Consequently, the external perception of virtual sounds depends on the degree of congruency between the acoustical features of the environment and the stimuli.

The impression of auditory space occurs on the basis of auditory cues provided by sound waves arriving at each ear, directly from the source, and after bouncing off the surfaces of the environment^1^,^2^. Time and intensity differences between the two ear signals determine, in most cases, the azimuthal localization of sounds^3^,^4^, whereas the perception of elevation is mainly associated with the direction-dependent filtering effect of the outer ear^5^. Distance perception has been shown to rely mostly on intensity, the ratio between the energy of direct and reflected sound, and the frequency content of the signal^6^,^7^,^8^,^9^. In an acoustic environment listeners are exposed to physical stimuli (sound events) that lead to perceived auditory images (auditory events). However, the same sound event can yield different auditory events due to cognitive factors and cross-modal processing^10^. For instance, the spatial impression can be affected by multisensory interaction, particularly between vision and hearing. Several studies indicated that there is a combined perception that considers inputs from the two sensory modalities^11^,^12^,^13^,^14^. This knowledge has been exploited in different applications, such as video gaming and multimedia reproduction in connection with virtual sound techniques that enable the generation of externalized sound images via headphones^10^,^15^, such that real-world sound sources are convincingly reproduced^16^,^17^.

Sound externalization refers to an out-of-head position for a given auditory event. Externalization can be defined as accurate when the auditory event is properly localized within a confined space in terms of distance and direction^18^. In contrast, internalization refers to an in-head auditory event position, with sound perceived between the ears or lateralized, without a projection of the auditory image in space^19^. This typically occurs during the reproduction of acoustic signals that have been simulated or recorded without considering the acoustic filtering due to diffraction from the pinna, head, and torso^20^. Such filtering is described by the head-related transfer function (HRTF), an accurate representation of which can enable listeners to perceive externalized sound images. Individualized HRTFs can be recorded from each listener. Alternatively, HRTFs can be synthesized or obtained from dummy heads, which results in decreased localization accuracy^21^. When the recording environment is not anechoic, the HRTFs also contain information about the acoustical properties of the environment and the corresponding impulse responses are called binaural room impulse responses (BRIRs)^10^,^22^. When the playback room and the room in which the BRIRs are recorded differ, the listener may receive incongruent room-related cues from the headphone reproduction and the listening environment^23^. Here, we aimed to test whether such incongruent room cues affect externalization accuracy, defined as the degree of coincidence between the virtually reproduced sound event and the perceived auditory event in terms of distance, direction, and compactness^18^.

In our experiments, we investigated whether the externalized perception during sound reproduction breaks down in certain environments where the spatial acoustic information from the playback signal and the cues obtained about the room are incongruent. We asked eighteen naive listeners to rate three spatial attributes of real sound sources (distance, azimuthal direction, and compactness) independently in order to evaluate sound externalization of virtual stimuli delivered via headphones. Although previous studies have addressed externalization through headphones as an overall percept^20^,^24^,^25^,^26^, none investigated which specific cues arising from the room are most important for externalization, and how they might be affected by a change of the listening environment.

Three rooms were used for the experiments. A standard IEC listening room^27^ was the *Reference* room for the listening test, in which individual BRIRs were recorded. As room acoustic parameters, such as reverberation time, are generally related to the volume of the room^28^, a smaller and larger room were also used in the listening tests. However, these rooms were acoustically treated such that the smaller room (*Reverberant-Small*) was much more reverberant and the larger room (*Dry-Large*) was anechoic, i.e., not reverberant at all. Thus, the incongruence between the spatial cues of the *Reference* and the other test rooms differed depending on whether the listener considered the room-related visual cues (i.e., difference in room volume) or auditory cues (i.e., difference in reverberation time).

For the listening experiment, the participants were divided into two groups and provided either auditory or visual awareness of the test rooms, while input from the other modality was limited as much as possible (Fig. 1a). One group of listeners could see the rooms but did not receive any auditory stimuli except the processed speech sentences. The other group entered the rooms blindfolded and performed the ratings in the dark, but was provided room-related auditory cues from a loudspeaker emitting noise bursts every 5 s. All subjects then performed the same experiment with both visual and auditory room cues available. The target signals were anechoic speech sentences convolved with BRIRs obtained for each listener individually before testing. All BRIRs were recorded for seven source positions in the reference room, while the test subjects wore both earplugs and blindfolds to avoid a priori knowledge of the room.

The listeners evaluated the three externalization attributes using subjective scales (Fig. 1b). Each stimulus was rated twice in each condition per room. Directional ratings were based on the selection of one out of twelve possible options arranged using a clock style notation. Compactness and distance perception were rated using a scale ranging from 0 to 5. For compactness, 0 corresponded to the most compact and 5 to the broadest perception of a sound. For distance, 0 indicated an auditory image perceived inside the head, 4 corresponded to a percept at the loudspeaker, and 5 beyond the loudspeaker position. During the experiments, four loudspeakers were visible at 0°, 30°, 90°, and 330° (XII, I, III, and XI o’clock, respectively, indicated by loudspeaker pictograms in Fig. 1b), while sounds were simulated for all seven recorded source positions (circled in red in Fig. 1b). An additional anechoic speech stimulus was presented diotically as a perceptual anchor, which was expected to be perceived inside the head due to the lack of spatial information in the signal^18^. The percepts from the simulation were compared to the percepts from a physical representation, which was achieved by delivering a randomly selected anechoic speech stimulus directly from the loudspeaker placed at III o’clock in the reference room. In the case of an ideal binaural simulation, this signal would be acoustically identical to the corresponding headphone signal and the two should be indistinguishable.

## Results

### Effect of mismatch between playback and recording room

In order to present the results using a similar metric for the three attributes of interest, a criterion for “correct” externalization was used. A “correct” response was defined as an auditory event perceived coincident in space with the physical sound event that would be produced by a loudspeaker at the corresponding position used for the BRIR recording. In order to take the limitations imposed by the virtual sound reproduction into account, the criterion was based on a comparison of listener ratings for headphone vs loudspeaker presentation. The ratings for position III showed that the anechoic signal played back through a loudspeaker in the reference room and the corresponding headphone signal yielded very similar distance ratings (Fig. S1a; Loudspeaker: *M* = 3.81, *SD* = 0.40; Headphones: *M* = 3.97, *SD* = 0.17) and azimuthal direction ratings (Fig. S1b; Loudspeaker: *M* = 3.00, *SD* = 0.00; Headphones: *M* = 2.97, *SD* = 0.17), whereas sounds delivered via headphones resulted in a wider range of compactness ratings compared to sounds presented from the loudspeaker (Fig. S1c; Loudspeaker: *M* = 0.22, *SD* = 0.42; Headphones: *M* = 1.11, *SD* = 1.12). Based on these results, the criterion for “correct” responses was defined as a score of 4 for distance, 0 to 1 for compactness, and localization at the exact azimuthal direction.

Figure 2 shows the percentages of correct responses for the three externalization parameters distance (Fig. 2a), azimuthal direction (Fig. 2b), and compactness (Fig. 2c). Ratings are shown using different colors for each playback room and are presented separately for the three conditions tested. The analysis for the condition in which both visual and auditory room cues were available (Fig. 2a, left) showed that sound externalization in terms of perceived distance is indeed room dependent. A linear mixed-effects-model analysis of variance (ANOVA) with Room, Cue, and Position as fixed factors and Listener as a random factor (Table S1) revealed a significant effect of Room [*F*(2,1456) = 94.6, *p* < 0.001]. Post hoc multiple comparisons using Tukey’s honest significant difference test also revealed significant differences across all playback rooms in the condition with both visual and auditory cues (Table S3, *p* < 0.001). Listeners generally perceived sounds to be closer to their heads in both incongruent rooms than in the reference room. The reverberant room (*Reverberant-Small*, blue bars) yielded the lowest externalization scores with approximately 18% of correct distance ratings. This confirms that a mismatch between recording and playback room adversely affects the externalization of binaural speech stimuli in terms of perceived distance.

Here, such a mismatch generally affected the distance ratings more for front and back positions than for lateral positions (Fig. 3), as reflected by a significant effect of Position [*F*(2,1456) = 28.8, *p* < 0.001] and a significant interaction between Room and Position [*F*(12,1456) = 4.9, *p* < 0.001] (Table S1). This was confirmed by significant differences for all post-hoc multiple comparisons between front-back (XII and VI) and lateral (III and IX) positions in the *Reverberant-Small* and *Dry-Large* rooms, while none of the differences between positions XII and VI (frontal) and between positions III and IX (lateral) were significant (Table S4). The post-hoc analysis also confirmed that the effect of room mismatch was more pronounced for the *Reverberant-Small* room, where it was significant for all positions except position III, than for the *Dry-Large* room, in which it reached significance only for positions XI and XII (Table S5). Finally, the distance ratings varied more across positions in the *Dry-Large* and *Reverberant-Small* rooms than in the *Reference* room (more significant between-Position differences in Table S4), and were overall lowest for sounds delivered from the VI o’clock position (Fig. 3a).

Unlike the distance ratings, the azimuthal direction (Fig. 2b, full bars) and compactness (Fig. 2c) judgements did not show a dependency on the listening environment. Instead, they varied with the position of the target stimuli (Figs 4 and 5), with the overall percentage of correct judgements ranging from 40 to 60%. Linear mixed-effects-model ANOVAs with Room, Cue, and Position as fixed factors and Listener as a random factor (Tables S6 and S9) confirmed that, for these two attributes, there was a significant effect of Position [Direction: *F*(6,700) = 40.6, *p* < 0.001; Compactness: *F*(6,1456) = 27.8, *p* < 0.001] but no effect of Room [Direction: *F*(2,700) = 0.4, *p* = 0.639; Compactness: *F*(2,1456) = 1.0, *p* = 0.357]. Lateral positions (III and IX) were consistently rated more accurately than front (XII) and back (VI) positions for both direction and compactness (Figs 4a and 5a). Post hoc multiple comparisons using Tukey’s honest significant difference test showed that, when all room cues were available, 7 out of 8 front/back vs lateral comparisons were significant, while ratings for positions III vs IX and VI vs XII never differed significantly (Tables S7 and S10).

For directional ratings (Fig. 4a), the best performance was observed for speech signals delivered from the III and IX o’clock positions in all rooms, whereas the worst performance was observed for positions VI, VII, and XII o’clock. The height of the empty bars in Fig. 2b indicates what the percentage of correct directional judgements would be if front-back confusions were considered as correct responses, i.e., the difference between empty and full bars reflects the rate of front-back confusions for each condition. A linear mixed-effects model ANOVA performed on the rate of front-back confusions revealed no significant effect of Room [*F*(2,700) = 0.6, *p* = 0.536] but a significant effect of Position [*F*(6,700) = 15.2, *p* < 0.001] (Table S12), and subsequent post-hoc analysis showed significant differences only for pairwise comparisons involving either positions VI or XII o’clock (Table S13). This confirms that listeners tended to localize peripheral sounds more easily, whereas front and back positions were often confused. In addition, the comparison of variance of the direction judgements showed a significant difference across positions (*χ*^2^(6) = 34.23, p < 0.001), with the lowest variance observed for lateral positions III and IX o’clock (Table S15). However, no clear effect was found of whether or not the stimuli were colocated with visible loudspeakers (Table S16).

In terms of compactness ratings (Fig 5a), the stimuli rated in all three environments showed a significantly larger reported source broadness for front and back than for lateral positions. The post-hoc analysis confirmed that, when both visual and auditory room cues were available, all front/back vs lateral comparisons were significantly different, while III vs IX and VI vs XII did not differ significantly (Table S7). Thus, virtual stimuli delivered from the front and back positions are not only problematic for sound localization, but also present a challenge for spatial segregation.

### Effect of auditory vs visual awareness of the room

The influence of the type of available room-related cues on sound externalization was studied by comparing the results for the condition where listeners received both visual and auditory room cues (Fig. 2, left panels) to the conditions where they received either visual (Fig. 2, middle panels) or auditory room cues (Fig. 2, right panels) only.

For azimuthal direction and compactness (Fig. 2b and c), no significant main effect was found between the tested conditions in the ANOVA [Direction: *F*(2,700) = 0.4, *p* = 0.673; Compactness: *F*(2,1456) = 0.7, *p* = 0.478] (Tables S6 and S9). There were significant interactions between Cue and Position for the two attributes [Direction: *F*(12,700) = 2.0, *p* = 0.019; Compactness: *F*(12,1456) = 2.6, *p* = 0.002]. However, the post-hoc analysis showed that none of the pairwise comparisons remained significant after correction for multiple comparisons for direction (Table S11), while only the comparison for all room cues vs visual room cues only in position XII remained significant for compactness (Table S8).

In contrast to the other two attributes, the pattern of distance judgements in the three rooms varied significantly between the three cue conditions (Fig. 2a). The ANOVA (Table S1) showed a significant effect of Cue [*F*(2,1456) = 7.6, *p* = 0.001], and a significant interaction between Room and Cue [*F*(4,1456) = 16.6, *p* < 0.001]. In the *Reference* room (Fig. 2a, red bars), the ratings obtained for conditions with either visual or auditory cues (middle and right panels) resulted in slightly lower distance ratings than in the condition with both cues available (left panel), but these differences were not significant (Table S2). The distance scores obtained in the *Dry-Large* room (Fig. 2a, green bars) were essentially unaffected by the type of cue, reflected by insignificant differences in judgements across conditions (Table S2). In contrast, in the *Reverberant-Small* room (Fig. 2a, blue bars), listeners were significantly more accurate (by 35%) in the visual room cue only condition (middle panel) compared to the other two conditions (left and right panels), which were similar. The post-hoc analysis confirmed that the presence of incongruent auditory room cues led to significantly lower distance ratings in the *Reverberant-Small* room (Fig. 2a, blue bars) only (*p* < 0.001, Table S2). Moreover, the differences in distance ratings between the *Reverberant-Small* and the other two rooms were only significant when auditory room cues were available (*p* < 0.001, Table S3). Therefore, the listeners’ distance judgements were reduced whenever they received auditory room cues from the playback room that did not match those from the recording room, while their judgements remained unaffected when they could see a room that differed from the one they heard through the headphone reproduction. This behavior was consistent across all source positions (Fig. 3b–d), and no interaction was found between Cue and Position in the ANOVA [*F*(12,1456) = 0.6, *p* = 0.818].

## Summary and Discussion

The above results indicate that a mismatch between the room in which the binaural headphone reproduction was played back and the room in which the BRIRs were recorded is detrimental to the externalization of the resulting auditory images in terms of their perceived distance. However, there was no evidence that such a room mismatch affects the perceived azimuthal direction or compactness of the auditory images.

The findings also suggest that the auditory modality has a higher impact on externalization in terms of perceived distance than the visual modality, when cues from the recording and playback room are incongruent. It should be noted that the perceptual judgements were obtained during an auditory-only task, which might explain why the observed effects only occurred when the information was incongruent within the same (auditory) modality and not across modalities. Concerning the role of the visual modality, a clear distinction should be made between room-related visual cues, which did not affect externalization here, and source-related visual cues, which have been reported to influence the auditory spatial impression in experiments where audiovisual stimuli convey spatial discrepancies^11^,^12^. In the present study, a statistical comparison of the variance of the direction estimates showed that it varied significantly across positions (Table S15). Although this reflects the fact that more confusions occurred for some positions than others (Fig. 4), the significant pairwise comparisons did not systematically occur between positions with and without visible loudspeakers (Table S16).

Overall, our results demonstrate that the highest degree of externalization is obtained in the presence of both auditory and visual congruent information. In incongruent listening situations, the auditory information about the room becomes more critical for the perception of distance when the listening environment is more reverberant compared to the recording room, but not when the listening room is anechoic. Such a result might be explained by the fact that the difference in reverberation time between the *Reference* and the *Reverberant-Small* room (2.4 s) was much larger than that between the *Reference* and the *Dry-Large* room (0.4 s). Moreover, it may also be due to the anechoic nature of the *Dry-Large* room. In a reverberant room, the only natural scenario in which a listener could hear an acoustic signal with comparatively low reverberation is if the sound source is very close. This might explain the lower distance ratings in the condition with all cues available in the *Reverberant-Small* room. However, in the anechoic *Dry-Large* room, the noise signal carried practically no room information, which might be the reason why the auditory incongruence did not result in conflicting room information and thus did not affect distance ratings in the *Dry-Large* room. In that sense, an anechoic room is a very special environment, and the results might well be different in a “real” room with a short but non-zero reverberation time.

The outcomes of the present study are relevant in listening experiments that use binaural stimuli, especially when the acoustical features of the listening environments differ from the inherent acoustic properties of the target signals. Therefore, special care should be taken during the selection of tests rooms, where matching acoustical features is more crucial than visual congruence. Considering this aspect may help reduce the bias of perceptual judgements in listening experiments using virtual headphone reproduction. In addition, our results suggest that the listening space of the user should be considered when designing virtual reality and multimedia reproduction systems.

## Methods

### Listeners and rooms

Eighteen naive subjects participated in the experiment (20–29 years old). None had been in any of the test rooms before. The subjects reported normal or corrected-to-normal vision; three of them wore corrective lenses. All had normal hearing, which was verified with pure tone audiograms obtained for each subject before testing. All subjects provided informed consent prior to their participation in the experiments, which were approved by the Science-Ethics Committee for the Capital Region of Denmark (reference H-3-2013-004) and carried out in accordance with the corresponding guidelines and relevant regulations on the use of human subjects for health-related scientific research. The experiments were conducted in three soundproof rooms, which were selected so that the acoustic features and dimensions contrast with each other. The reference room (*Reference*) had a reverberation time of 0.4 s and a volume of 99 m^3^. The other two listening rooms (*Reverberant-Small* and *Dry-Large*) had reverberation times of 2.8 s and <0.01 s, and volumes of 43.2 m^3^ and 330.4 m^3^, respectively. The background noise level was below 19 dB(A) in all three rooms.

### BRIR recordings

**Figure 1:**
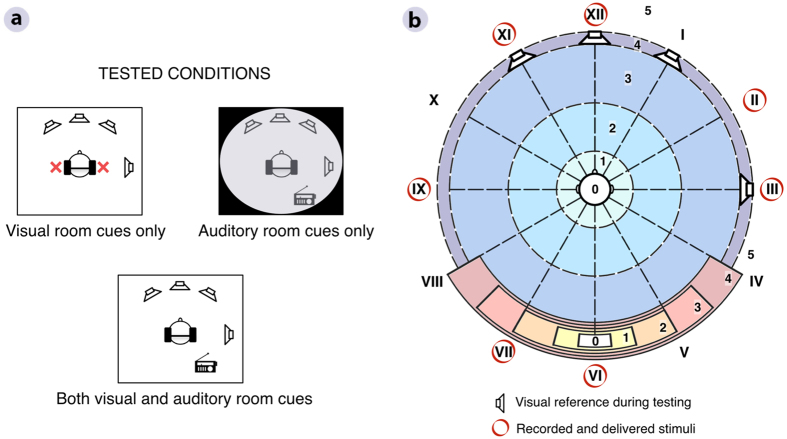
Experimental conditions and setup. (**a**) Illustration of the three experimental conditions: visual room cues, auditory room cues, and both visual and auditory room cues. (**b**) Loudspeaker setup and subjective rating scales used in the experiments. For azimuthal direction judgements, listeners could provide ratings from I to XII. For distance and compactness judgements, listeners could provide ratings from 0 to 5.

For the BRIR recordings, the listeners were instructed to keep as quiet as possible while they were in the *Reference* room. Blindfolds and earplugs reduced the available visual or auditory information about the room and the loudspeakers. The listeners were seated on a listening chair looking straight ahead. A headrest was provided to help them keep their heads still. Omnidirectional DPA 4060 lapel microphones were placed at the ear canal entrance, on top of the earplugs, and attached to the pinna with a wire hook. Seven azimuthal source positions were recorded at a distance of 1.5 m from the listener: 0°, 60°, 90°, 180°, 210°, 270°, and 330°, also referred to as positions XII, II, III, VI, VII, IX, and XI o’clock, respectively (red circles in Fig. 1b). These were selected to provide front-back positions (XII and VI), within cone of confusion positions (II, VII, and XI), and lateral, outside cone of confusion positions (III and IX).

Six repetitions of a 5-s logarithmic sine sweep per position were reproduced through Dynaudio BM6P loudspeakers placed at eye level. The BRIRs were then obtained using a deconvolution method^29^. At the listener position, the sound pressure level measured with a B&K 2250 sound level meter was 65 dB(A). The recordings and playback were made through a portable M-audio Fast Track Ultra sound card at a sampling frequency of 48 kHz. Sennheiser HD 800 headphones were used in the listening experiment. To compensate for their effect in the transmission path, individual headphone impulse responses (HPIRs) were recorded. To do so, ten 2-s logarithmic sine sweeps were played back through the headphones positioned on the listeners while the microphones were still placed in the same position. As before, the resulting HPIRs were then transformed into the frequency domain using the fast Fourier Transform. A regularization parameter was used to remove the frequency content of the headphone responses below 50 Hz and above 18 kHz. The speech material, the BRIRs, and HPIRs were then convolved, and the resulting signals were stored to be used during the experiment. After this procedure was completed, the test subject was guided outside the room, where earplugs and blindfolds were removed.

### Stimuli

The stimuli were male speech sentences with a duration of approximately 2 s each. In total, 24 different sentences were taken from the Danish version of the hearing in noise test (HINT)^30^. Eight different signals were used in each room, seven were convolved with the BRIRs, and one unprocessed signal was presented diotically through the headphones (Anchor). In the reference room one additional signal was reproduced from the loudspeaker positioned at III o’clock. This was done to inspect whether results were different between real (loudspeaker) and virtual (headphones) stimuli, and thus verify the accuracy of the binaural reproduction. The results obtained were used to define the criteria for correct ratings that were regarded as those with a score of 4 for distance, localized at the correct azimuthal direction, and within the range 0 to 1 for compactness. To ensure that the headphones did not unduly attenuate the sounds from the room, real-ear insertion gains were measured with probe microphones for one listener. The result for a frontal loudspeaker position showed that the headphones caused some attenuation between about 1.5 and 5.3 kHz. The average attenuation across this range was −5.7 dB, the maximum attenuation occurred as a dip of 8.9 dB at 2.66 kHz. While this causes some minor coloration of the acoustic scene, it is still clearly possible to assess the characteristics of the room through the headphones.

### Experimental procedure

The order of the rooms in the listening experiment was randomly determined for each listener. Each participant was assigned a group that defined the starting condition, either visual room cues only or auditory room cues only. Listeners from the first group entered the first room seeing the environment, but listening to loud music over headphones (85 dB SPL approx.). The subjects were also instructed to avoid speaking and to keep as quiet as possible while they were in the room. Once in the listening position, the music was stopped and the listeners started the experiment. Participants from the second group entered the room wearing blindfolds but no earplugs. Once seated, the lights were turned off and they were allowed to uncover the eyes. The light provided by the user interface (iPad) was sufficient to see the loudspeaker positions but no further into the room. In addition, small dimmed lights were placed on top of each loudspeaker to ensure that listeners always had a clear visual reference for the position. To provide auditory room cues, white noise bursts of 500-ms duration were reproduced. The noise signal was played back through a Bose Soundlink Mobile speaker located behind the listener (at V o’clock). The distance from the test subject was 2 m and the sound pressure level at the listening position was 35 dB(A), which was well below the stimulus level and therefore assumed not to distract from the experimental task. Given the unfamiliar nature of the noise and its low level, listeners were able to segregate this signal from the target speech stimuli delivered over headphones. All listeners were also instructed to keep their head still and look at the front loudspeaker during stimulus presentation, but the head was not fixated, because the externalization percept seemed fairly robust with respect to small head movements. Once the starting condition was completed, the participants took a short break outside the room. Then, in the same room, the listeners performed the experiment in condition with both visual and auditory room cues (i.e., without any visual or auditory restrictions). In this condition, the external noise source was also activated. The procedure was repeated in the other two rooms in random order. The listeners wore the headphones during the whole experiment and while entering the test rooms for the group with visual room cues only. They did not wear headphones while being guided from one room to the next.

The listening ratings were done through an iPad user interface implemented in MATLAB R2015a, where the subjects could push buttons to rate the different externalization parameters. A training session was conducted in the very first trial of the initial condition to familiarize the listeners with the task. The training comprised one complete experimental run for all three attributes and lasted about 10–15 min. No feedback was given on the ratings, but a short discussion was held to make sure that the attributes were understood correctly. In each condition the experiment was divided into two blocks. In the first block, listeners were first asked to judge the perceived direction of the stimuli by selecting that one of the twelve possible numbers on a clock style notation, which best represented the direction of the incoming sound (Fig. 1b). Once a direction was chosen and confirmed, the same stimulus was presented again, but this time a compactness rating was required. For this attribute, the test subjects were provided an interface containing concentric coloured areas with increasing broadness. Six options were available, where area 0 was the most compact perception, corresponding to the area occupied by the loudspeaker. Area 5, on the other hand, represented a compactness perception that exceeded 120°. Once the compactness rating was selected and confirmed, a new signal was delivered randomly at another position. The rest of the experimental block was completed by interleaving the ratings for the two parameters. In the second block, distance judgements were obtained for the same stimuli. Subjects were presented with a diagram containing six concentric zones with increasing diameter, where zone 0 represented perception inside the head, zone 4 a sound perceived as coming from the loudspeaker position, and zone 5 a stimulus perceived at a distance beyond the loudspeaker position. Zones 1, 2, and 3 represented the source being perceived at the ears, at a location closer to the listener than the loudspeaker, and at a location closer to the loudspeaker than the listener, respectively. Two ratings were obtained at each position tested for all the three parameters. A replay button enabled the subjects to repeat the stimuli as often as required.

Four loudspeakers placed at positions I, III, XI, and XII o’clock (loudspeaker pictograms in Fig. 1b) were visible during the test. These were labelled accordingly. The loudspeaker setup provided a visual reference during the experiment and served to study the potential influence of visual targets on auditory perception, especially for virtual stimulus positions adjacent to loudspeakers.

### Statistical analysis

**Figure 2:**
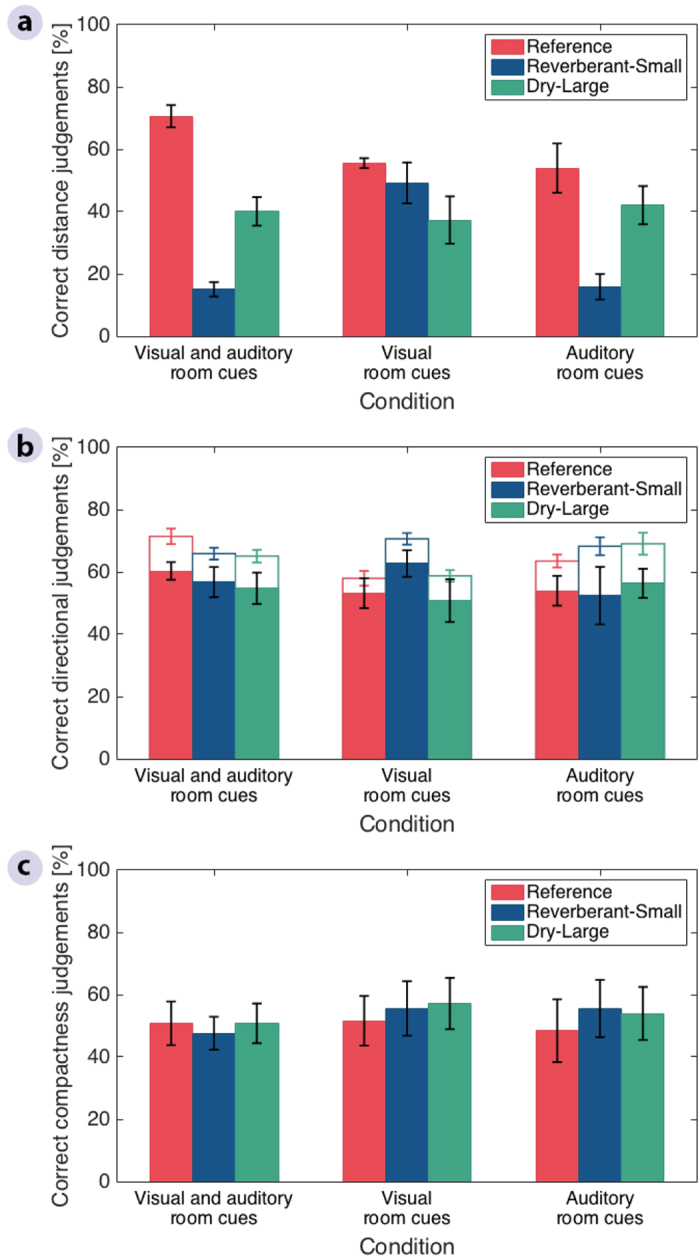
Total correct judgements of each externalization parameter in the reference room and the two rooms that were incongruent with the reference. (**a**) Correct distance ratings. (**b**) Correct azimuthal direction ratings. (**c**) Correct compactness ratings. Percentages represent the across-listener mean calculated over the total number of correct judgements per listener across positions, while error bars show the standard error of the mean across listeners. In (**b**) filled bars represent percentages of correct directional judgements, while the height of empty bars represent the same percentages when counting front-back confusions as correct. Front-back confusions were determined from the number judgements in a hemisphere that differed from that of the stimulus position over the total number of presentations.

**Figure 3:**
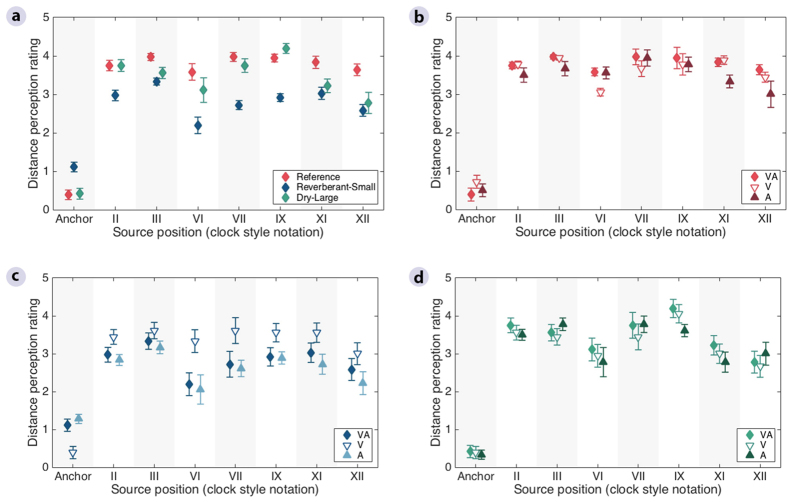
Average distance perception ratings in the reference room and the two rooms that were incongruent with the reference, as a function of source position. The ratings were obtained in three conditions, with both visual and auditory room cues (VA), and with either visual (V) or auditory room cues only (A). (**a**) Condition with both visual and auditory room cues across the three rooms. (**b**) Three tested conditions in the *Reference* room. (**c**) Three tested conditions in the *Reverberant-Small* room. (**d**) Three tested conditions in the *Dry-Large* room. The average of the two presentations per listener was used to calculate the across-listener mean, and the error bars show the standard error of the mean across listeners.

**Figure 4:**
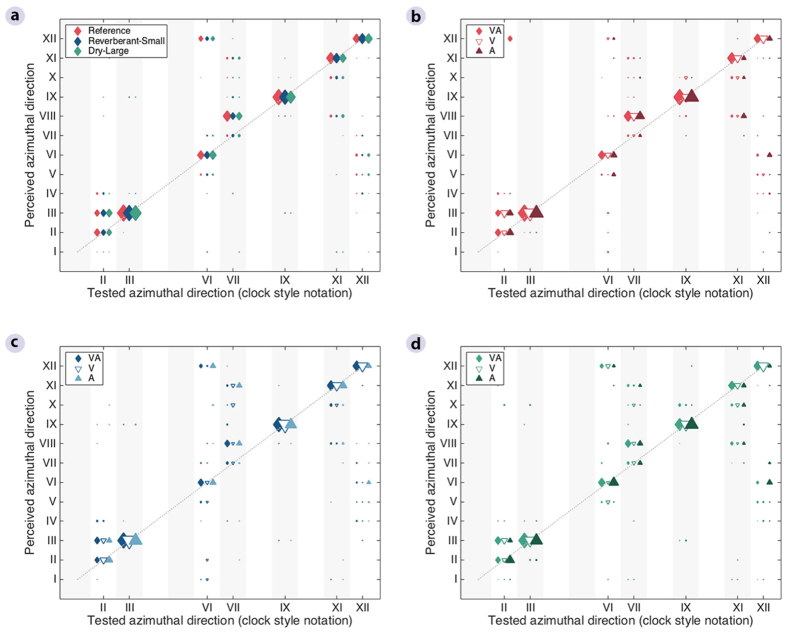
Azimuthal direction ratings in the reference room and the two rooms that were incongruent with the reference, as a function of source position. Marker size reflects the number of responses for each tested vs. perceived direction. The ratings were obtained in three conditions, with both visual and auditory room cues (VA), and with either visual (V) or auditory room cues only (A). (**a**) Condition with both visual and auditory room cues across the three rooms. (**b**) Three tested conditions in the *Reference* room. (**c**) Three tested conditions in the *Reverberant-Small* room. (**d**) Three tested conditions in the *Dry-Large* room.

**Figure 5:**
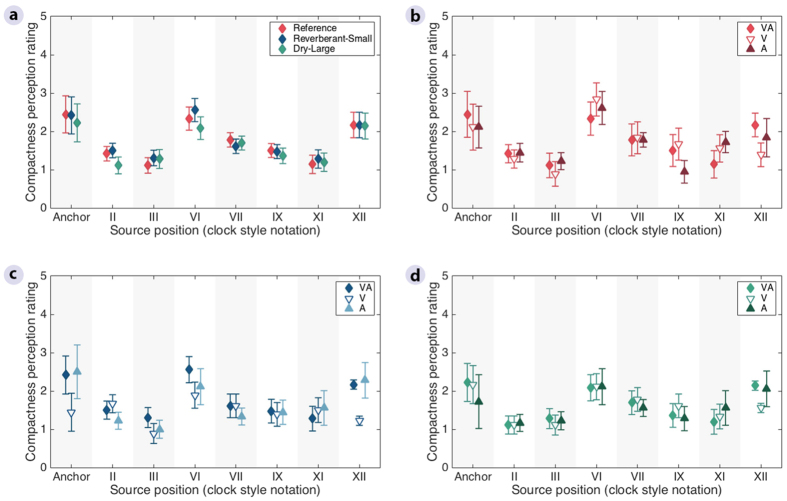
Average compactness perception ratings in the reference room and the two rooms that were incongruent with the reference, as a function of source position. The ratings were obtained in three conditions, with both visual and auditory room cues (VA), and with either visual (V) or auditory room cues only (A). (**a**) Condition with both visual and auditory room cues across the three rooms. (**b**) Three tested conditions in the *Reference* room. (**c**) Three tested conditions in the *Reverberant-Small* room. (**d**) Three tested conditions in the *Dry-Large* room. The average of the two presentations per listener was used to calculate the across-listener mean, and the error bars show the standard error of the mean across listeners.

A significance level of 0.05 was used for all analyses. The statistical analyses for distance and compactness data were performed on the raw listener ratings (summarized in Figs 3 and 5), before transformation to the percent correct ratings shown in Fig. 2. Although the underlying subjective attributes for distance and compactness could be assumed to vary on continuous scales, the collected data was ordinal in nature due to the use of discrete rating scales, for which the assumption of equally-distant scale points was not necessarily valid. In order to test whether a parametric linear mixed-effects model could be robust enough to the non-continuous nature of the data and whether this would increase the risk of Type I errors, one thousand data sets were simulated by keeping Listener, Room, Cue, and Position the same as in the original data set but taking each listener’s distance and compactness values to be a random sample from their original distance and compactness values with replacement. The resulting alpha-values were very close to 0.05, indicating that the Type I error rate was not unduly increased and that a linear mixed-effects model could be assumed to be robust enough to the non-continuous dependent variables.

A linear mixed-effects model with Room, Cue, and Position as fixed factors, and Listener as a random factor was fitted to the data. Visual inspection of the residuals showed no major deviation from normality or homoscedasticity. The statistical analysis for distance and compactness ratings was thus based on an ANOVA (Tables S1 and S6). A reduced model, from which insignificant three-way interactions were removed, was used. A post-hoc analysis using Tukey’s honest significant difference test was carried out to study multiple pairwise comparisons when both main and interaction effects were significant (Tables S2 to S5, S7 and S8).

For azimuthal direction, mixed-effects ANOVA models similar to that used for the analysis of distance and compactness judgements was used, except that the analyses were performed on the rate of correct judgements (Table S9) and the rate of front-back confusions (Table S12) presented in Fig. 2b. Front-back confusions were calculated by dividing the number of judgements in a hemisphere that differed from that of the stimulus position over the number of presentations. Post-hoc pairwise comparisons were again studied using Tukey’s honest significant difference test (Tables S10, S11, S13, and S14).

The variance of directional ratings pooled across conditions for each position and subject was compared across positions using a Friedman test (Table S15) and pairwise comparisons were studied with Bonferroni-corrected Wilcoxon sign-rank tests (Table S16).

## Additional Information

**How to cite this article**: Gil-Carvajal, J.C. *et al.* Spatial Hearing with Incongruent Visual or Auditory Room Cues. *Sci. Rep.* **6**, 37342; doi: 10.1038/srep37342 (2016).

**Publisher’s note:** Springer Nature remains neutral with regard to jurisdictional claims in published maps and institutional affiliations.

## Supplementary Material

Supplementary Information
