# Supplementary material for: Spatial Hearing with Incongruent Visual or Auditory Room Cues
Source: Sci Rep. 2016 Nov 17;6:37342. doi: 10.1038/srep37342 (PMC5112595; doi:10.1038/srep37342)
Supplement: Supplementary Information [file srep37342-s1.doc]

**Spatial Hearing with Incongruent Visual or Auditory Room Cues**

*Juan C. Gil Carvajal, Jens Cubick, Sébastien Santurette, & Torsten Dau*

**Supplementary information**

**Supplementary data: Comparison of loudspeaker and headphone presentation**

**Figure S1:** Distributions of listeners’ judgements for each externalization parameter for anechoic stimuli presented through the loudspeaker (black bars) vs the corresponding headphone signal (grey bars). The ratings were obtained in the *Reference* room for stimuli delivered from position III with both visual and auditory room cues available. **a)** Distribution of distance judgements. Means and standard deviations: Headphones (*M* = 3.97, *SD* = 0.17); Loudspeaker (*M* = 3.81, *SD* = 0.40). **b)** Distribution of directional judgements. Means and standard deviations: Headphones (*M* = 2.97, *SD* = 0.17); Loudspeaker (*M* = 3.00, *SD* = 0.00). **c)** Distribution of compactness judgements. Means and standard deviations: Headphones (*M* = 1.11, *SD* = 1.11); Loudspeaker (*M* = 0.22, *SD* = 0.42). Distance and compactness judgements were averaged per subject over the two trials. Only ratings that had an occurrence above 0% are presented.

**
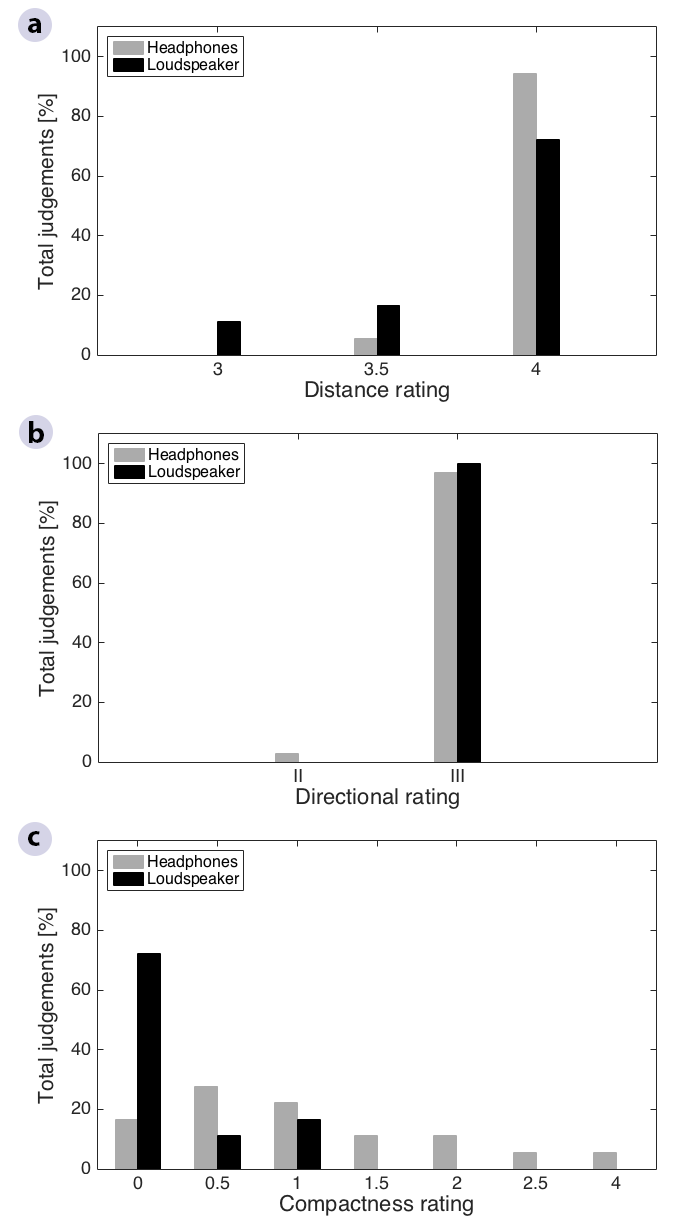
**

**Statistical analysis: Detailed results**

The results of the statistical analyses are detailed in the following tables. Note that for distance and compactness perception all statistical tests were performed on the raw ratings data and not the percent correct outcomes presented in Fig. 2. For perceived azimuthal direction, the tests were performed on both the rate of correct judgements and the rate of front-back confusions. In the following tables, VA stands for “Visual and Auditory room cues”, V for “Visual room cues only”, and A for “Auditory room cues only”. ***: *p* < .0010; **: *p* < .0100; *: *p* < .0500.

**Table S1:** Statistical analysis for perceived distance ratings. Results of a linear mixed-effects model ANOVA with Room, Cue, and Position as fixed factors and Listener as random factor. A full model showed a non-significant three-way interaction, such that a reduced model including two-way interactions only was used.

**numDF denDF *F*-value *p*-value**

**Room**  2 1456 94.6338 <.0001***

**Cue**  2 1456 7.6176 0.0005***

**Position**  6 1456 28.7569 <.0001***

**Room:Cue**  4 1456 16.6246 <.0001***

**Room:Position**  12 1456 4.8822 <.0001***

**Cue:Position**  12 1456 0.6296 0.8184

**Table S2:** Post-hoc analysis for perceived distance ratings. *t*-ratios and *p*-values (in brackets) of pairwise contrasts averaged over positions for the Cue factor as a function of Room, using Tukey’s honest significant difference test.

**Reference room Reverberant-Small Dry-Large**

**VA vs V** 1.975 (0.5611) -6.989 (<.0001***) 2.156 (0.4353)

**VA vs A** 3.005 (0.0670) 0.822 (0.9962) 1.737 (0.7233)

**V vs A** 0.822 (0.9962) 7.109 (<.0001***) -0.334 (1.0000)

**Table S3:** Post-hoc analysis for perceived distance ratings. *t*-ratios and *p*-values (in brackets) of pairwise contrasts averaged over positions for the Room factor as a function of Cue, using Tukey’s honest significant difference test. Ref: Reference room; Rev: Reverberant-Small room; Dry: Dry-Large room.

**VA V A**

**Ref vs Rev** 14.521 (<.0001***) 2.136 (0.4492) 9.282 (<.0001***)

**Ref vs Dry** 4.879 (<.0001***) 3.614 (0.0094**) 2.300 (0.3427)

**Rev vs Dry** -9.642 (<.0001***) 1.479 (0.8656) -6.982 (<.0001***)

**Table S4:** Post-hoc analysis for perceived distance ratings. *t*-ratios and *p*-values (in brackets) of pairwise contrasts averaged over cue conditions for the Position factor as a function of Room, using Tukey’s honest significant difference test.

**Reference Room Reverberant-Small Dry-Large**

**II vs III** -1.541 (0.9949) -2.395 (0.6895) 0.379 (1.0000)

**II vs VI** 1.861 (0.9570) 4.636 (0.0007***) 4.956 (0.0002***)

**II vs VII** -1.601 (0.9918) 0.960 (1.0000) -0.427 (1.0000)

**II vs IX** -1.257 (0.9997) -0.083 (1.0000) -2.857 (0.3377)

**II vs XI** -0.095 (1.0000) -0.095 (1.0000) 4.600 (0.0009***)

**II vs XII** 2.099 (0.8745) 3.592 (0.0474*) 6.473 (<.0001***)

**III vs VI** 3.403 (0.0858) 7.031 (<.0001***) 4.576 (0.0010**)

**III vs VII** -0.059 (1.0000) 3.355 (0.0987) -0.806 (1.0000)

**III vs IX** 0.285 (1.0000) 2.312 (0.7490) -3.237 (0.1377)

**III vs XI** 1.446 (0.9977) 2.300 (0.7571) 4.221 (0.0046**)

**III vs XII** 3.640 (0.0405*) 5.987 (<.0001***) 6.094 (<.0001***)

**VI vs VII** -3.462 (0.0717) -3.675 (0.0359*) -5.383 (<.0001***)

**VI vs IX** -3.118 (0.1875) -4.719 (0.0005***) -7.813 (<.0001***)

**VI vs XI** -1.956 (0.9311) -4.731 (0.0005***) -0.356 (1.0000)

**VI vs XII** 0.237 (1.0000) -1.043 (1.0000) 1.518 (0.9958)

**VII vs IX** -0.884 (1.000) -1.043 (1.0000) -2.430 (0.6627)

**VII vs XI** 1.506 (0.9962) -1.055 (1.0000) 5.027 (0.0001***)

**VII vs XII** 3.699 (0.0331*) 2.632 (0.5046) 6.900 (0.0001***)

**IX vs XI** 1.162 (0.9999) -0.012 (1.0000) 7.457 (0.0001***)

**IX vs XII** 3.355 (0.0987) 3.675 (0.0359*) 9.331 (0.0001***)

**XI vs XII** 2.193 (0.8243) 3.687 (0.0345*) 1.873 (0.9543)

**Table S5:** Post-hoc analysis for perceived distance ratings. *t*-ratios and *p*-values (in brackets) of pairwise contrasts averaged over cue conditions for the Room factor as a function of Position, using Tukey’s honest significant difference test. Ref: Reference room; Rev: Reverberant-Small room; Dry: Dry-Large room.

**II III VI**

**Ref vs Rev** 4.389 (0.0022**) 3.527 (0.0585) 7.193 (<.0001***)

**Ref vs Dry** 0.370 (1.0000) 2.310 (0.7502) 3.496 (0.0645)

**Rev vs Dry** -4.020 (0.0102*) -1.217 (0.9998) -3.696 (0.0334*)

**VII IX XI**

**Ref vs Rev** 6.977 (<.0001***) 5.575 (<.0001***) 4.389 (0.0022**)

**Ref vs Dry** 1.556 (0.9942) -1.248 (0.9997) 5.113 (0.0001***)

**Rev vs Dry** -5.421 (<.0001***) -6.823 (<.0001***) 0.724 (1.0000)

**XII**

**Ref vs Rev** 5.899 (<.0001***)

**Ref vs Dry** 4.790 (0.0004***)

**Rev vs Dry** -1.109 (1.0000)

**Table S6:** Statistical analysis for perceived compactness ratings. Results of a linear mixed-effects model ANOVA with Room, Cue, and Position as fixed factors and Listener as random factor. A full model showed a non-significant three-way interaction, such that a reduced model including two-way interactions only was used.

**numDF denDF *F*-value *p*-value**

**Room**  2 1456 1.03099 0.3569

**Cue**  2 1456 0.73583 0.4780

**Position**  6 1456 27.83419 <.0001***

**Room:Cue**  4 1456 0.76010 0.5513

**Room:Position**  12 1456 0.98721 0.4587

**Cue:Position**  12 1456 2.58324 0.0021**

**Table S7:** Post-hoc analysis for perceived compactness ratings. *t*-ratios and *p*-values (in brackets) of pairwise contrasts averaged over rooms for the Cue factor as a function of Position, using Tukey’s honest significant difference test.

**VA V A**

**II vs III** 0.783 (1.0000) 1.938 (0.9368) 0.646 (1.0000)

**II vs VI** -6.917 (<.0001***) -4.614 (0.0008**) -4.983 (0.0001***)

**II vs VII** -2.480 (0.6247) -1.938 (0.9368) -1.384 (0.9988)

**II vs IX** -0.718 (1.0000) -1.015 (1.0000) 0.277 (1.0000)

**II vs XI** 0.979 (1.0000) -0.554 (1.0000) -1.661 (0.9873)

**II vs XII** -5.742 (<.0001***) -0.185 (1.0000) -3.876 (0.0176)

**III vs VI** -7.700 (<.0001***) -6.552 (<.0001***) -5.629 (<.0001***)

**III vs VII** -3.263 (0.1282) -3.876 (0.0176) -2.030 (0.9044)

**III vs IX** -1.501 (0.9963) -2.953 (0.2761) -0.369 (1.0000)

**III vs XI** 0.196 (1.0000) -2.492 (0.6154) -2.307 (0.7523)

**III vs XII** -6.526 (<.0001***) -2.123 (0.8627) -4.522 (0.0012**)

**VI vs VII** 4.437 (0.0018**) 2.676 (0.4701) 3.599 (0.0463)

**VI vs IX** 6.199 (<.0001***) 3.599 (0.0463*) 5.260 (<.0001***)

**VI vs XI** 7.896 (<.0001***) 4.061 (0.0087**) 3.322 (0.1085)

**VI vs XII** 1.175 (0.9999) 4.430 (0.0019**) 1.107 (1.0000)

**VII vs IX** 1.762 (0.9756) 0.923 (1.0000) 1.661 (0.9873)

**VII vs XI** 3.459 (0.0725) 1.384 (0.9988) -0.277 (1.0000)

**VII vs XII** -3.263 (0.1282) 1.753 (0.9768) -2.492 (0.6154)

**IX vs XI** 1.697 (0.9839) 0.461 (1.0000) -1.938 (0.9368)

**IX vs XII** -5.025 (0.0001**) 0.831 (1.0000) -4.153 (0.0060**)

**XI vs XII** -6.721 (<.0001***) 0.369 (1.0000) -2.215 (0.8117)

**Table S8:** Post-hoc analysis for perceived compactness ratings. *t*-ratios and *p*-values (in brackets) of pairwise contrasts averaged over rooms for the Position factor as a function of Cue, using Tukey’s honest significant difference test.

**II III VI**

**VA vs V** -0.178 (1.0000) 1.387 (0.9987) 0.135 (1.0000)

**VA vs A** 0.492 (1.0000) 0.596 (1.0000) 0.387 (1.0000)

**V vs A** 0.558 (1.0000) -0.659 (1.0000) 0.210 (1.0000)

**VII IX XI**

**VA vs V** -0.387 (1.0000) -0.752 (1.0000) -1.587 (0.9926)

**VA vs A** 0.909 (1.0000) 1.379 (0.9988) -2.170 (0.8377)

**V vs A** 1.079 (1.0000) 1.775 (0.9736) -0.485 (1.0000)

**XII**

**VA vs V** 4.205 (0.0049**)

**VA vs A** 0.700 (1.0000)

**V vs A** -2.919 (0.2975)

**Table S9:** Statistical analysis for the rate of correct directional judgements. Results of a linear mixed-effects model ANOVA with Room, Cue, and Position as fixed factors and Listener as random factor. A full model showed a non-significant three-way interaction, such that a reduced model including two-way interactions only was used.

**numDF denDF *F*-value *p*-value**

**Room**  2 700 0.44859 0.6387

**Cue**  2 700 0.39694 0.6725

**Position**  6 700 40.61668 <.0001***

**Room:Cue**  4 700 1.09885 0.3560

**Room:Position**  12 700 0.61351 0.8318

**Cue:Position**  12 700 2.03975 0.0189*

**Table S10:** Post-hoc analysis for the rate of correct directional judgements. *t*-ratios and *p*-values (in brackets) of pairwise contrasts averaged over rooms for the Cue factor as a function of Position, using Tukey’s honest significant difference test.

**VA V A**

**II vs III** -7.359 (<.0001***) -4.930(0.0002***) -3.469 (0.0716)

**II vs VI** -1.420 (0.9982) 0.913 (1.0000) 0.183 (1.0000)

**II vs VII** 3.098 (0.1986) 1.826 (0.9642) 3.104 (0.1960)

**II vs IX** -6.068 (<.0001***) -3.834 (0.0213*) -3.286 (0.1218)

**II vs XI** -2.711 (0.4443) -1.461 (0.9974) 1.278 (0.9996)

**II vs XII** -2.582 (0.5445) -2.739 (0.4236) 1.826 (0.9642)

**III vs VI** 5.939 (<.0001***) 5.842 (<.0001***) 3.652 (0.0399*)

**III vs VII** 10.457 (<.0001***) 6.755 (<.0001***) 6.573 (<.0001***)

**III vs IX** 1.291 (0.9995) 1.095 (1.0000) 0.183 (1.0000)

**III vs XI** 4.648 (0.0008***) 3.469 (0.0716) 4.747 (0.0005***)

**III vs XII** 4.777 (0.0004***) 2.191 (0.8251) 5.295 (<.0001***)

**VI vs VII** 4.519 (0.0014**) 0.913 (1.0000) 2.921 (0.2974)

**VI vs IX** -4.648 (0.0008***) -4.747 (0.0005***) -3.469 (0.0716)

**VI vs XI** -1.291 (0.9995) -2.374 (0.7049) 1.095 (1.0000)

**VI vs XII** -1.162 (0.9999) -3.652 (0.0399) 1.643 (0.9886)

**VII vs IX** -9.166 (<.0001***) -5.660 (<.0001***) -6.390 (<.0001***)

**VII vs XI** -5.810 (<.0001***) -3.286 (0.1218) -1.826 (0.9642)

**VII vs XII** -5.680 (<.0001***) -4.564 (0.0011**) -1.278 (0.9996)

**IX vs XI** 3.357 (0.0999) 2.374 (0.7049) 4.564 (0.0011**)

**IX vs XII** 3.486 (0.0680) 1.095 (1.0000) 5.112 (0.0001***)

**XI vs XII** 0.129 (1.0000) -1.278 (0.9996) 0.548 (1.000)

**Table S11:** Post-hoc analysis for the rate of correct directional judgements. *t*-ratios and *p*-values (in brackets) of pairwise contrasts averaged over rooms for the Position factor as a function of Cue, using Tukey’s honest significant difference test.

**II III VI**

**VA vs V** -0.072 (1.0000) 0.239 (1.0000) 2.105 (0.8706)

**VA vs A** -1.587 (0.9925) 0.383 (1.0000) -0.239 (1.0000)

**V vs A** 1.271(0.9996) -0.120 (1.0000) 1.966 (0.9272)

**VII IX XI**

**VA vs V** -0.486 (1.0000) 0.447 (1.0000) 0.447 (1.0000)

**VA vs A** -0.550 (1.0000) -0.447 (1.0000) 2.041 (0.8993)

**V vs A** 0.054 (1.0000) 0.749 (1.0000) -1.337 (0.9992)

**XII**

**VA vs V** -1.108 (1.0000)

**VA vs A** 2.559 (0.5623)

**V vs A** -3.076 (0.2096)

**Table S12:** Statistical analysis for the rate of front-back confusions. Results of a linear mixed-effects model ANOVA with Room, Cue, and Position as fixed factors and Listener as random factor. A full model showed a non-significant three-way interaction, such that a reduced model including two-way interactions only was used.

**numDF denDF *F*-value *p*-value**

**Room**  2 700 0.62513 0.5355

**Cue**  2 700 1.47168 0.2302

**Position**  6 700 15.20517 <.0001***

**Room:Cue**  4 700 0.67401 0.6101

**Room:Position**  12 700 0.58808 0.8528

**Cue:Position**  12 700 2.29771 0.0071**

**Table S13:** Post-hoc analysis for the rate of front-back confusions. *t*-ratios and *p*-values (in brackets) of pairwise contrasts averaged over rooms for the Cue factor as a function of Position, using Tukey’s honest significant difference test.

**VA V A**

**II vs III** 2.327 (0.7383) 1.371 (0.9989) 0.274 (1.0000)

**II vs VI** -3.684 (0.0358*) -0.548 (1.0000) -3.565 (0.0531)

**II vs VII** -0.776 (1.0000) -1.919 (0.9416) -3.016 (0.2412)

**II vs IX** 2.327 (0.7383) 1.371 (0.9989) 0.274 (1.0000)

**II vs XI** 2.133 (0.8568) 1.371 (0.9989) -0.548 (1.0000)

**II vs XII** -0.969 (1.0000) 0.823 (1.0000) -4.661 (0.0007**)

**III vs VI** -6.011 (<.0001***) -1.919 (0.9416) -3.839 (0.0209*)

**III vs VII** -3.102 (0.1968) -3.290 (0.1204) -3.290 (0.1204)

**III vs IX** 0.000 (1.0000) 0.000 (1.0000) 0.000 (1.0000)

**III vs XI** -0.194 (1.0000) 0.000 (1.0000) -0.823 (1.0000)

**III vs XII** -3.296 (0.1185) -0.548 (1.0000) -4.936 (0.0002**)

**VI vs VII** 2.908 (0.3055) -1.371 (0.9989) 0.548 (1.0000)

**VI vs IX** 6.011 (<.0001***) 1.919 (0.9416) 3.839 (0.0209*)

**VI vs XI** 5.817 (<.0001***) 1.919 (0.9416) 3.016 (0.2412)

**VI vs XII** 2.714 (0.4417) 1.371 (0.9989) -1.097 (1.0000)

**VII vs IX** 3.102 (0.1968) 3.290 (0.1204) 3.290 (0.1204)

**VII vs XI** 2.908 (0.3055) 3.290 (0.1204) 2.468 (0.6339)

**VII vs XII** -0.194 (1.0000) 2.742 (0.4211) -1.645 (0.9885)

**IX vs XI** -0.194 (1.0000) 0.000 (1.0000) -0.823 (1.0000)

**IX vs XII** -3.296 (0.1185) -0.548 (1.0000) -4.936 (0.0002***)

**XI vs XII** -3.102 (0.1968) -0.548 (1.0000) -4.113 (0.0074**)

**Table S14:** Post-hoc analysis for the rate of correct localisation judgements. *t*-ratios and *p*-values (in brackets) of pairwise contrasts averaged over rooms for the Cue factor as a function of Position, using Tukey’s honest significant difference test.

**II III VI**

**VA vs V** 0.246 (1.0000) -0.068 (1.0000) 2.602 (0.5286)

**VA vs A** 1.638 (0.9890) 0.068 (1.0000) 0.539 (1.0000)

**V vs A** -1.187 (0.9999) -0.116 (1.0000) 1.759 (0.9756)

**VII IX XI**

**VA vs V** -1.324 (0.9993) -0.068 (1.0000) 0.089 (1.0000)

**VA vs A** -1.189 (0.9999) 0.068 (1.0000) -0.717 (1.0000)

**V vs A** -0.116 (1.0000) -0.116 (1.0000) 0.688 (1.0000)

**XII**

**VA vs V** 1.974 (0.9246)

**VA vs A** -2.916 (0.3005)

**V vs A** 4.170 (0.0059**)

**Table S15:** Statistical analysis of the variance of directional judgements as a function of position. Results of a Friedman test carried out over the variances calculated per subject per position.

**Mean rank**

**II** 3.50

**III** 2.08

**VI** 5.39

**VII** 4.89

**IX** 2.97

**XI** 4.28

**XII** 4.89

**N** 18

**Chi-square (**
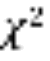
**)** 34.230

**df** 6

**Asymp. sig.** < 0.0001

**Table S16:** Post-hoc analysis on the variance of directional judgements as a function of position. Pairwise comparisons using Wilcoxon signed rank tests with Bonferroni corrections (significance indicated by * for *p* < 0.0023).

***p*-value**

**II vs III** 0.002*

**II vs VI** 0.007

**II vs VII** 0.007

**II vs IX** 0.177

**II vs XI** 0.033

**II vs XII** 0.011

**III vs VI** 0.003

**III vs VII** 0.002*

**III vs IX** 0.233

**III vs XI** 0.001*

**III vs XII** 0.004

**VI vs VII** 0.016

**VI vs IX** 0.004

**VI vs XI** 0.031

**VI vs XII** 0.407

**VII vs IX** 0.001*

**VII vs XI** 0.744

**VII vs XII** 0.112

**IX vs XI** 0.009

**IX vs XII** 0.006

**XI vs XII** 0.109
